# Functional Programming of the Autonomic Nervous System by Early Life Immune Exposure: Implications for Anxiety

**DOI:** 10.1371/journal.pone.0057700

**Published:** 2013-03-06

**Authors:** Luba Sominsky, Erin A. Fuller, Evgeny Bondarenko, Lin Kooi Ong, Lee Averell, Eugene Nalivaiko, Peter R. Dunkley, Phillip W. Dickson, Deborah M. Hodgson

**Affiliations:** 1 Laboratory of Neuroimmunology, School of Psychology, The University of Newcastle, Callaghan, New South Wales, Australia; 2 School of Biomedical Sciences and Pharmacy and the Hunter Medical Research Institute, The University of Newcastle, Callaghan, New South Wales, Australia; 3 Newcastle Cognition Laboratory, School of Psychology, The University of Newcastle, Callaghan, New South Wales, Australia; Université de Montréal, Canada

## Abstract

Neonatal exposure of rodents to an immune challenge alters a variety of behavioural and physiological parameters in adulthood. In particular, neonatal lipopolysaccharide (LPS; 0.05 mg/kg, i.p.) exposure produces robust increases in anxiety-like behaviour, accompanied by persistent changes in hypothalamic-pituitary-adrenal (HPA) axis functioning. Altered autonomic nervous system (ANS) activity is an important physiological contributor to the generation of anxiety. Here we examined the long term effects of neonatal LPS exposure on ANS function and the associated changes in neuroendocrine and behavioural indices. ANS function in Wistar rats, neonatally treated with LPS, was assessed via analysis of tyrosine hydroxylase (TH) in the adrenal glands on postnatal days (PNDs) 50 and 85, and via plethysmographic assessment of adult respiratory rate in response to mild stress (acoustic and light stimuli). Expression of genes implicated in regulation of autonomic and endocrine activity in the relevant brain areas was also examined. Neonatal LPS exposure produced an increase in TH phosphorylation and activity at both PNDs 50 and 85. In adulthood, LPS-treated rats responded with increased respiratory rates to the lower intensities of stimuli, indicative of increased autonomic arousal. These changes were associated with increases in anxiety-like behaviours and HPA axis activity, alongside altered expression of the GABA-A receptor α2 subunit, CRH receptor type 1, CRH binding protein, and glucocorticoid receptor mRNA levels in the prefrontal cortex, hippocampus and hypothalamus. The current findings suggest that in addition to the commonly reported alterations in HPA axis functioning, neonatal LPS challenge is associated with a persistent change in ANS activity, associated with, and potentially contributing to, the anxiety-like phenotype. The findings of this study reflect the importance of changes in the perinatal microbial environment on the ontogeny of physiological processes.

## Introduction

Activation of the immune system in early life is thought to play a role in predisposing to later life psychopathologies. Epidemiological evidence, for instance, indicates higher incidences of psychopathologies, including schizophrenia, negative emotionality and panic disorders in children exposed to viral or bacterial infections in-utero [1], [2] or in early life [3]. Animal models that have been utilised to examine the impact of immune activation in early life have repeatedly demonstrated that exposure to bacterial or viral agents including mimetics (e.g. lipopolysaccharide (LPS); polyinosinic:polycytidylic acid (poly I:C)), live agents (e.g. *Escherichia coli (E. coli)*), and other agents, such as toxins (i.e., endotoxins and exotoxins) is associated with an increased likelihood of psychopathology in later life [4], [5], [6], [7], [8], [9].

LPS exposure on postnatal days (PNDs) 3 and 5 is a well-documented rodent model used to examine the impact of “perinatal programming” on a variety of physiological and behavioural outcomes [4], [5], [6], [10], [11], [12], [13], [14], [15], [16], [17]. The concept of perinatal programming encompasses the role of the intrauterine and early postnatal environment in the onset of adult disease [18]. One of the most consistent observations is that exposure to LPS in early life results in offspring who, in adulthood, demonstrate increased anxiety-like behaviours [4], [13], [15]. The anxiety-like behaviours typically observed include more time spent in the closed arms and fewer entries to the open arms of an elevated plus maze (EPM), reduced exploratory behaviour in the holeboard apparatus and increased risk assessment behaviour in the open field apparatus [4], [13], [15]. Importantly, recent evidence indicates that these behavioural outcomes are not limited to the exposed animals alone, but have been shown to persist into a subsequent generation of offspring, born to either a maternal or paternal line of LPS-treated animals [6].

The system most commonly implicated in the development of anxiety-like behaviours, in the context of neonatal exposure to LPS, is the hypothalamic-pituitary-adrenal (HPA) axis. Alterations in circulating corticosterone have been reported in adult animals treated as neonates with LPS. This is particularly apparent when these animals are subjected to an additional acute stressor in adulthood [4], [11], [12], [16]. At the central level, neonatal exposure to LPS has been shown to be associated with increased corticotropin-releasing hormone (CRH) mRNA levels in the hypothalamus in male rats and decreased glucocorticoid receptor (GRs) density in the hypothalamus, hippocampus and frontal cortex of both male and female rats [11]. Exposure to live infection in early life was also reported to produce long term alterations in HPA axis activity, with increased plasma corticosterone levels in adult males and increased GR mRNA levels in the hippocampus of adult females, when compared to same sex controls [19]. Overall, it has been suggested that early life exposure to an inflammatory insult alters the later response of the HPA or stress axis. Whilst the HPA axis response to a peripheral inflammatory challenge is primarily mediated by the proinflammatory cytokines, such as interleukin (IL)-1β, tumor necrosis factor (TNF)-α and IL-6 [20], further evidence suggests a possible role of γ-aminobutyric acid (GABA) in LPS-induced activation of the HPA axis [21]. GABAergic innervation of the hypothalamus provides an inhibitory tone regulating the HPA axis activity [22]. Administration of GABA prior to LPS injection has been shown to reduce plasma corticosterone levels when compared to animals treated with LPS alone [21]. Nevertheless, increased activation of GABA-A receptors in neonatal rats increases hippocampal neuronal apoptosis, resulting in memory and learning impairments in later life [23]. Of particular relevance, neonatal LPS exposure on PNDs 7 and 9 has been recently demonstrated to result in a selective decrease of GABA containing interneurons in the hippocampal regions of adult rats [24], indicating altered GABA signalling induced by neonatal immune activation.

The HPA axis is not, however, the only system affected by neonatal exposure to an inflammatory insult. The HPA axis and the autonomic nervous system (ANS) work in a coordinated fashion to modulate the stress response. Recent evidence from our laboratory indicates that neonatal exposure of rodent pups to LPS results in an immediate and sustained response of the ANS as indicated by an increase in phosphorylation and activity of tyrosine hydroxylase (TH) in the adrenal glands [13], [14], [25]. Given the critical role of TH in catecholaminergic synthesis, this finding suggests that neonatal LPS treatment results in an increased activation of the sympathetic compartment of the ANS.

Exposure to LPS has been demonstrated to induce activation of the ANS during the acute-phase immune response. Specifically, parasympathetic nervous system signalling was evidenced via both the afferent and efferent vagal signals. Peripheral administration of LPS was reported to increase levels of circulating catecholamines and to cause a distinct activation of central catecholaminergic neurons that are likely to play a mediating role in the neuroendocrine responses to peripheral inflammation (reviewed in [26], [27]). Moreover, whilst intraperitoneal administration of LPS led to an increase in noradrenergic, serotonergic and dopaminergic activity in a range of brain areas, intracerebroventricular (i.c.v.) administration of the bacterial mimetic did not have an effect on dopamine metabolism suggesting involvement of other factors induced by peripherally administered LPS [28].

ANS activation during the LPS-induced inflammatory response promotes HPA axis activity, facilitating physiological adaptation to the immunological challenge [27]. Given this evidence it is possible that exposure to neonatal LPS may alter the long term functioning of the ANS. To date, however, relatively little is known regarding the programming effect of early life exposure to LPS on ANS functioning. Our recent findings have indicated increased TH phosphorylation in the adrenal glands of LPS-treated neonates, in both males [13], [25] and females [14]. The aim of the current study was to determine whether neonatal LPS exposure is able to induce long term alterations in autonomic activity. To assess this we utilised both a direct measure of autonomic activity (i.e., adrenal TH) and a behavioural measure of autonomic function (i.e. respiratory response as measured by plethysmography).

Respiratory rate is a sensitive and reliable index of autonomic arousal, independent of changes in cardiovascular parameters. Whilst cardiovascular measures are most commonly utilised, the use of plethysmography (a non-invasive technique able to measure respiration) facilitates the measurement of changes in respiration at thresholds far lower than that required to induce autonomically mediated changes in cardiovascular system [29], [30]. Therefore, assessment of respiratory rate in rodents provides an index of the orienting response, constitutive of ANS arousal [30]. Moreover, in humans, anxiety states are often associated with respiratory dysregulation [31].

In the current study neonatal programming of the ANS was investigated via the assessment of TH activation in the adolescent and adult adrenal glands. Autonomic function was further validated by the measurement of respiratory responses to mild stressful stimuli in adult rats. To confirm the presence of an anxiety-like phenotype, adult anxiety-like behaviours were evaluated, and HPA axis activity at both the peripheral and central levels was assessed. Brain factors examined included mRNA levels of CRH, CRH binding protein (CRHBP), CRH receptor type 1 (CRHR1), GRs and mineralocorticoid receptors (MRs) as well as GABA-A receptor subunit alpha-2 (GABA-Arα2), due to its role in modulation of the stress response and anxiety states [32].

## Methods

### Experimental Procedures

**Figure 1:**
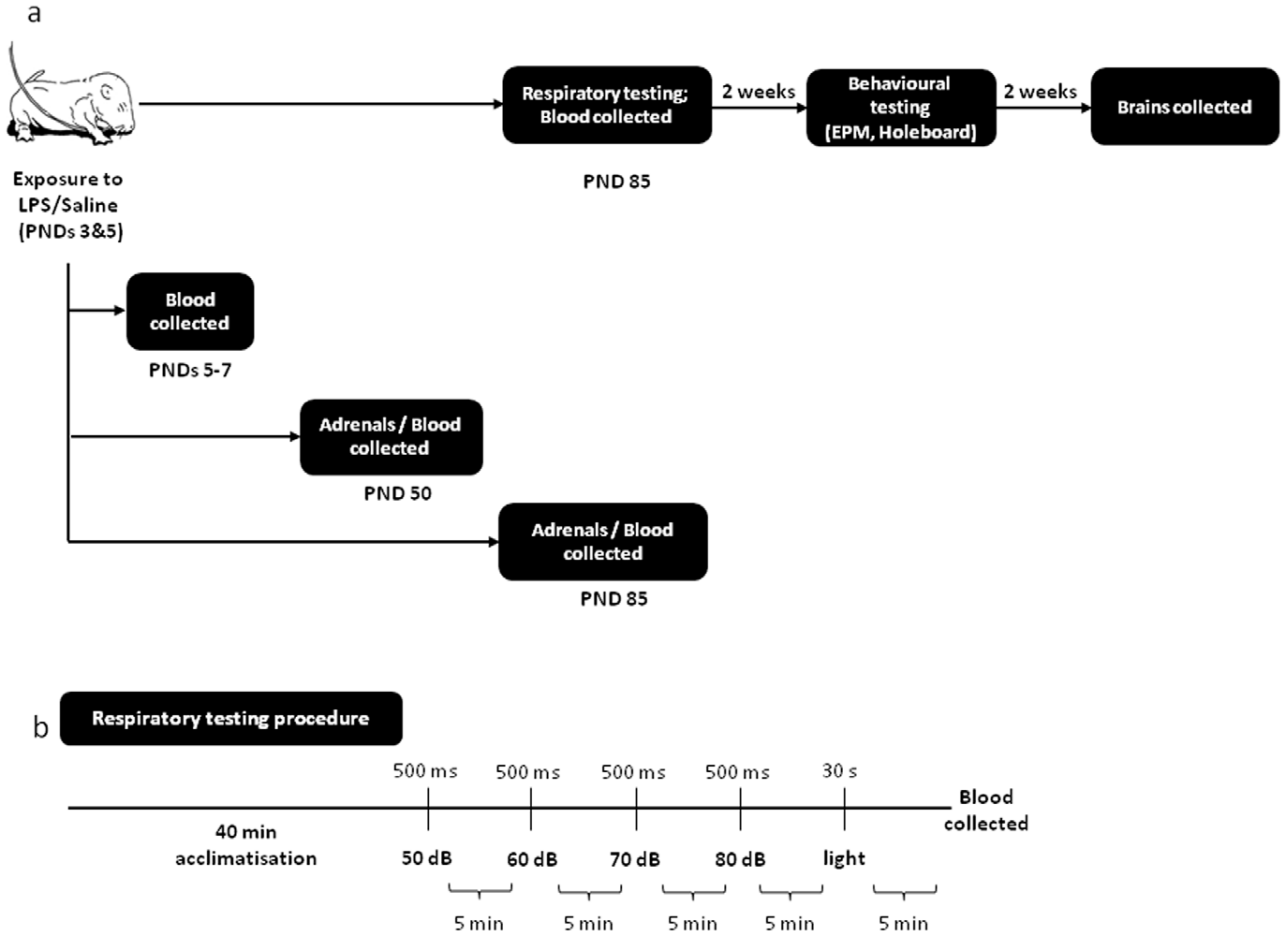
Experimental design. (A) A schematic timeline of the study design and experimental protocols. (B) A schematic representation of the respiratory testing procedure.

Experimentally naive female Wistar rats were obtained from the University of Newcastle animal house and mated in the University of Newcastle Psychology vivarium, under conventional housing conditions. At birth (PND 1), pups were randomly allocated into either LPS or saline control conditions. Pups were briefly removed from their cages on PNDs 3 and 5, weighed and administered with LPS (Salmonella enterica, serotype enteritidis; Sigma-Aldrich Chemical Co., USA, dissolved in sterile pyrogen-free saline, 0.05 mg/kg) or an equivolume of saline (Livingstone International, Australia), intraperitoneally (i.p.). Neonatal drug administration protocols and housing conditions were previously reported [4], [17]. Only male offspring were used in this study. A subgroup of 47 males (derived from four LPS and four saline-treated litters) was euthanized at 4 h, 24 h and 48 h post neonatal treatment on PND 5 to determine the efficacy of drug administration via assessment of plasma corticosterone levels (n = 6−10 per group, per time point). The remaining pups were housed with their dams until PND 22, at which point they were weaned and divided into same-sex housing (41.5 × 28.0 × 22.0 cm cages; Mascot Wire Works, Sydney Australia) and left undisturbed except for weekly weights and monitoring. In adolescence (PND 50) an additional subgroup of animals (16 males, derived from three LPS and two saline-treated litters; n = 6−10 per group) was sacrificed for analysis of plasma corticosterone and adrenal TH levels. In adulthood (PND 85), 10 LPS and 9 Saline treated male rats (derived from three LPS and two saline-treated litters) were subjected to respiratory and behavioural testing, as further described. Brains were collected from 13 animals (n = 5−7 per group) two weeks following the behavioural testing to determine expression of CRH, CRHR1, CRHBP, GABA-Arα2, GR and MR mRNA levels. An additional group of 6 LPS and 6 saline-treated males (derived from two LPS and two saline-treated litters) were used for assessment of baseline plasma corticosterone and TH levels in adulthood. See the study design in Fig. 1a. For each experimental condition, animals were distributed as evenly as possible from all litters used per treatment, to avoid potential litter effects. All experiments were conducted in accordance with the 2004 NH&MRC Australian Code of Practice for the care and use of animals for scientific practice. This study was approved by The University of Newcastle Animal Care and Ethics Committee (ACEC 901).

### Blood and Adrenals Collection

#### Neonatal time point

At 4 h, 24 h and 48 h following the last neonatal drug administration on PND 5, animals were rapidly decapitated and trunk blood was collected into EDTA-coated tubes (Livingstone International, Australia).

#### Adolescence

On PND 50 animals were deeply anesthetized with an overdose of Lethabarb (2 ml/kg i.p.; Virbac, Pty. Ltd, Milperra, Australia). Cardiac blood was obtained into EDTA-coated tubes. Adrenals glands were surgically removed, cleaned of any excess fat tissue, immediately frozen in liquid nitrogen and kept at −80°C until assayed.

#### Adulthood

On PND 85, immediately following the respiratory testing blood was collected via the saphenous vein. All blood samples were centrifuged at 1000 × g for 15 min at 4°C, and plasma stored at −20°C until assayed. Blood and adrenal glands were collected from an additional subset of animals on PND 85, after a lethal injection of Lethabarb (2 ml/kg), following the previously described methods.

### Radioimmunoassay

Plasma corticosterone concentrations were assessed using a commercially available rat corticosterone 125I radioimmunoassay kit (MP Biomedicals, USA). The recovery of free corticosterone is 100%, with an inter- and intra-assay variability of 4.4% and 6.5%, respectively.

### Behavioural Testing

Anxiety-like behaviours in adulthood were assessed on the elevated plus maze (EPM) and the holeboard apparatus, as per previously described protocols [4], [6], [16]. Animals were given 5 min to explore each of the apparatuses and the order of testing was counterbalanced. Behaviours assessed in the EPM included the percentage of time spent in the open arms, number of entries to the open and closed arms, and risk assessment. Anxiety-related variables examined in the holeboard apparatus included exploratory head dips and time spent in the centre square. Activity and distance travelled were assessed in both the EPM and the holeboard, for indications of locomotor activity and freezing. Time and event-related data for the assessment of anxiety-like behaviours and locomotor activity were recorded using a computer-based automated behavioural tracking system (Motion Mensura Pty. Ltd., Australia). The computer software allows a definition of regions of interest and monitors entries to and time spent in these regions, using pixel intensity to determine the position of the animal and general locomotor activity.

### Tissue Collections and Quantitative RT-PCR

Expression of CRH, CRHR1, CRHBP and GABA-Arα2 mRNA levels was determined by quantitative RT-PCR in the prefrontal cortex (PFC), hypothalamus and hippocampus of LPS and saline-treated animals. In addition, expression of GRs and MRs was determined in the hypothalamus and hippocampus. Two weeks following the respiratory testing, animals were deeply anesthetized with an overdose of Lethabarb (2 ml/kg i.p.; Virbac, Pty. Ltd, Milperra, Australia), brains were rapidly removed and areas of interest isolated (PFC, hypothalamus and hippocampus). Samples were then immersed in RNA*later* solution (Ambion, Austin, TX, USA), stored at 4°C overnight and then kept at −20°C until further analysis.

Total RNA extraction from brain tissue was carried out using RNeasy Lipid Tissue Mini Kit (Qiagen Inc., Valencia, CA, USA) according to the manufacturer’s instructions. RNA concentrations were determined by spectrophotometer, NanoDrop 2000c (Thermo Fisher Scientific, Wilmington, DE USA). First-strand cDNA was synthesized from 2 µg of total RNA using a SuperScript® VILO™ cDNA Synthesis Kit (Invitrogen Corp., Carlsbad, CA, USA), according to manufacturers’ instructions. Real-time PCR was performed using SYBR Green PCR Master Mix (Invitrogen, Carlsbad, CA, USA) on a 7500 RT-PCR Fast instrument (Applied Biosystems, Foster City, CA, USA). Primer sequences are listed in Table 1. The 25 µl PCR mixture consisted of 12.5 µl SYBR Green PCR Master Mix, 9.5 µl water and 2 µl of each primer was added to 1 µl of the cDNA template (10 ng/ml). All reactions were performed in duplicate under the following conditions: 95°C for 20 s and 40 cycles of 95°C for 3 s and 60°C for 30 s. In addition, a melting curve was determined under the following conditions: 95°C for 15 s, 60°C for 1 min, 95°C for 15 s and 60°C for 15 s. The data were normalized to an endogenous control, β-actin. A relative quantitative measure of the target gene expression compared with β-actin mRNA was obtained using the equation 2*^−ΔΔC(t)^*, where *C(t)* is the cycle at which fluorescence was first detected above background, and presented as a fold increase relative to the saline control.

**Table 1:** Real-time PCR primer details.

Target gene	Forward	Reverse
*CRH*	CGCCCATCTCTCTGGATCTC	CGTTGTAAAGTAAAGGGCTATTAG
*CRHR1*	TGGAACCTCATCTCGGCTTT	CACTCGACCTGGTGTTTGGT
*CRHBP*	GCGAAGGCGAGGGAAGAA	GTACCGACCGGAACACAGA
*GABA-A-rα2*	CAATGCACTTGGAGGACTTTCC	GGCTCCAGCACATTCGTATCG
*GR*	CGTCAAAAGGGAAGGGAAC	TGTCTGGAAGCAGTAGGTAAG
*MR*	CCAAATCACCCTCATCCAG	GCACAGTTCATACATGGCAG
*β-actin*	TCTGTGTGGATTGGTGGCTCTA	GACGAACGACTAGGTGTAGAC

### Tyrosine Hydroxylase Analysis

Tyrosine hydroxylase phosphorylation, protein and activity levels were analysed as previously described with some modification [25]. Briefly, the adrenals were homogenised using a sonicator (Soniprep 150, MSE) in 500µL homogenisation buffer (50 mM Tris-HCl pH 7.5, 1 mM EGTA, 1 protease tablet, 1 mM Sodium Vanadate, 1 mM Sodium Pyrophosphate, 80µM Ammonium Molybdate, 5 mM β-Glycerophosphate, 2 µM Microcystin). Samples were then centrifuged at 16,000 rpm for 20 min at 4°C. The clear supernatants were collected and protein concentration was determined by a BCA assay according to the manufacturer’s general protocol for protein analysis. Samples were diluted with homogenisation buffer to same concentration (5 mg/mL).

Thirty µg of each sample was mixed with sample buffer (1% SDS, 10% glycerol, 0.5% DTT and minimal bromophenol blue) and subjected to SDS–polyacrylamide gel electrophoresis before being transferred to nitrocellulose. Membranes were incubated with blocking solution (5% bovine serum albumin) for 2 h at 25°C. Membranes were washed in Tris-buffered saline with Tween (TBST) (150 mM NaCl, 10 mM Tris, 0.075% Tween-20, pH 7.5) and incubated with pSer19-, pSer31-, pSer40-, total-TH or β-actin-HRP antibodies for 1 h at 25°C. Membranes were washed in TBST and incubated with HRP-linked anti-IgG secondary specific antibodies for 1 h at 25°C. Membranes were visualized on Fugifilm Las-3000 imaging system (Fuji, Stamford, CT, USA) using Western HRP Substrate (Millipore). The densities of phospho-, total TH and β-actin bands were measured using a MultiGauge V3.0 (Fuji, Stamford, CT, USA). Total TH protein levels were expressed as the ratio of TH protein to β-actin as β-actin levels are used as an endogenous control. pSer19, pSer31 and pSer40 were expressed as the ratio to total TH protein to account for variability in total TH between samples.

Fifty µg of each sample was then mixed with reaction mixture (36 µg catalase, 2 mM potassium phosphate pH 7.4, 0.008% β-mercaptoethanol, 24 µM L-tyrosine, 1 µCi 3,5-[^3^H]-L-tyrosine, final volume 50 µL) and subjected to tritiated water release assay. The 50 µL reactions were initiated with the addition of 100 µM tetrahydrobiopterin in 5 mM HCl. Control representing background reactions were added with 5 mM HCl but did not contain tetrahydrobiopterin. Assays were performed for 20 min at 30°C and were stopped by addition of 700 µL charcoal slurry (7.5% activated charcoal in 1 M HCl). Mixtures were vortexed for 1 min and were centrifuged at 16,000 rpm for 10 min at 30°C. 350 µL supernatants were added to 3 mL scintillation cocktail and were vortexed for 10 s. Mixtures were assayed by scintillation spectrometry for 10 min per sample. TH activity assays which were performed under these conditions were linear. The changes in TH activity were normalized to total TH protein levels and expressed as a fold increase relative to the saline control.

### Respiratory Testing

#### Materials

Respiratory rate was recorded using a custom-built whole body plethysmograph, based on the procedure previously described [29], [30]. Briefly, the apparatus consisted of a clear Perspex cylinder (inner diameter 95 mm, length 260 mm, volume 1.84 l, wall thickness 3 mm), flushed by a constant flow of compressed medical air (3 L/min). The output flow was separated into two lines by a plastic T-connector, with one line connected to a differential pressure amplifier (model 24PC01SMT, Honeywell Sensing and Control, GoldenValley,MN,USA) and the second line open to room air. Air pressure signal from the amplifier was digitised at 1 KHz and collected via a data acquisition system (PowerLab, Model 4SP, ADInstruments, Sydney, Australia). Respiratory rate was computed online from the pressure signal using ChartPro 6.0 software (AD Instruments, Sydney, Australia). A piezoelectric pulse transducer (MLT1010/D, AD Instruments, Sydney, Australia) was located underneath the plethysmograph, to record gross motor activity.

#### Procedure

All recordings occurred in a dark chamber. As illustrated in Fig. 1b, following 40 min acclimatisation to the cylinder, animals were presented with four increasing intensities of acoustic stimuli in ascending order (50 dB, 60 dB, 70 dB, 80 dB). Each acoustic stimulus was presented for 500 ms with an inter-stimulus interval of 5 min. The light stimulus (30 Lux, 30 s duration) was presented 5 min after the last acoustic stimulus.

#### Analysis

Each baseline period was defined as ten respiratory cycles in duration with no motor activity preceding the presentation of the stimulus. In cases where motor activity was present, data was excluded from further analysis. Respiratory response to a stimulus was then determined as a maximal change in respiratory rate for a duration of at least two respiratory cycles. Hierarchical Bayesian analysis, based on Markov Chain Monte Carlo (MCMC) methods [33] was employed to evaluate changes in respiratory rate in response to increasing intensities of acoustic stimuli. The MCMC implementation was carried out in WinBUGS [34]. A Change Detection mathematical model was developed to assess the presence and magnitude of changes in respiratory rate in response to amplification of low-intensity acoustic stimuli among LPS and saline-treated animals. Thus, hierarchical modelling allowed us to determine whether the experimental groups vary in their propensity to physiological arousal, avoiding the bias associated with averaging data and or the noise associated with analysis of individual cases. Here we model the sequence of respiratory recordings as a normal distribution where each trial (recording) is estimated by the evaluation of the likelihood that the current posterior distribution and the proposed distribution are from the same sequence or not. When the latter is more likely, a new distribution is proposed and evaluated; iteratively the proposal distribution (prior) settles to a new posterior distribution at a new mean level, with equal variance assumed for each proposed distribution. The fixing of the variance parameter allows the mean estimates to be dependent upon the largest change in the sequence.

For each stimulus intensity (50 dB, 60 dB, 70 dB, 80 dB) changes in respiratory rate were presented as a difference (Δ values) attained by subtracting the baseline respiratory rate prior to the onset of stimulus from the peak respiratory response to the stimulus, as generated by the Change Detection model. Student *t* test analyses on Δ respiratory rate were performed and complemented by Bayesian *t* tests, presented as posterior odds of preference for either null *(H_0_*) versus alternative hypotheses (*H_1_*) (for additional information on Bayes factor analysis see [35]). Bayes factor for *H_0_* versus *H_1_* is presented as *B*_01_.

### Data Analysis

Statistical analyses were conducted using the Statistical Package for the Social Sciences for Windows, Version 18 (SPSS Inc.). All data, except for the changes in respiration in response to acoustic stimuli (as described above), were analysed using analyses of variances (ANOVA) design. A nested ANOVA design, whereby “litter” is nested into “treatment” and analyses of covariance (ANCOVA), whereby “male-to-female ratio” and “litter size” are covaried in the analyses, were also employed where appropriate and reported only when significantly contributing to the data. Planned comparisons were performed when significant interactions were observed using *t* test analyses adjusted for multiple comparisons. The significance level was set at *p*≤0.05.

## Results

### Developmental Weight Gain

Weight gain was recorded in all males born into the litters participating in this study. No differences in weight gain were observed between the LPS and saline-treated animals during the neonatal period. However, LPS-treated males gained significantly more weight, than saline-treated controls (*F*_(1,89)_ = 26.925, *p*<.001) following weaning (PND 22) to adolescence (PND 50). Significant weight gain in LPS treated animals, as compared to saline-treated controls, was also evident from adolescence (PND 50) to adulthood (PND 85) (*F*_(1,58)_ = 17.623, *p*<.001). See Table 2.

**Table 2:** Developmental Weight Gain.

Developmental timeframe	LPS	Saline
*PND 22–50*	213.52±5.23***	176.13±4.95
*PND 50–85*	241.55±11.99***	174.71±10.48

Values are mean±SEM.

*** *p*<.001.

### Plasma Corticosterone

**Figure 2:**
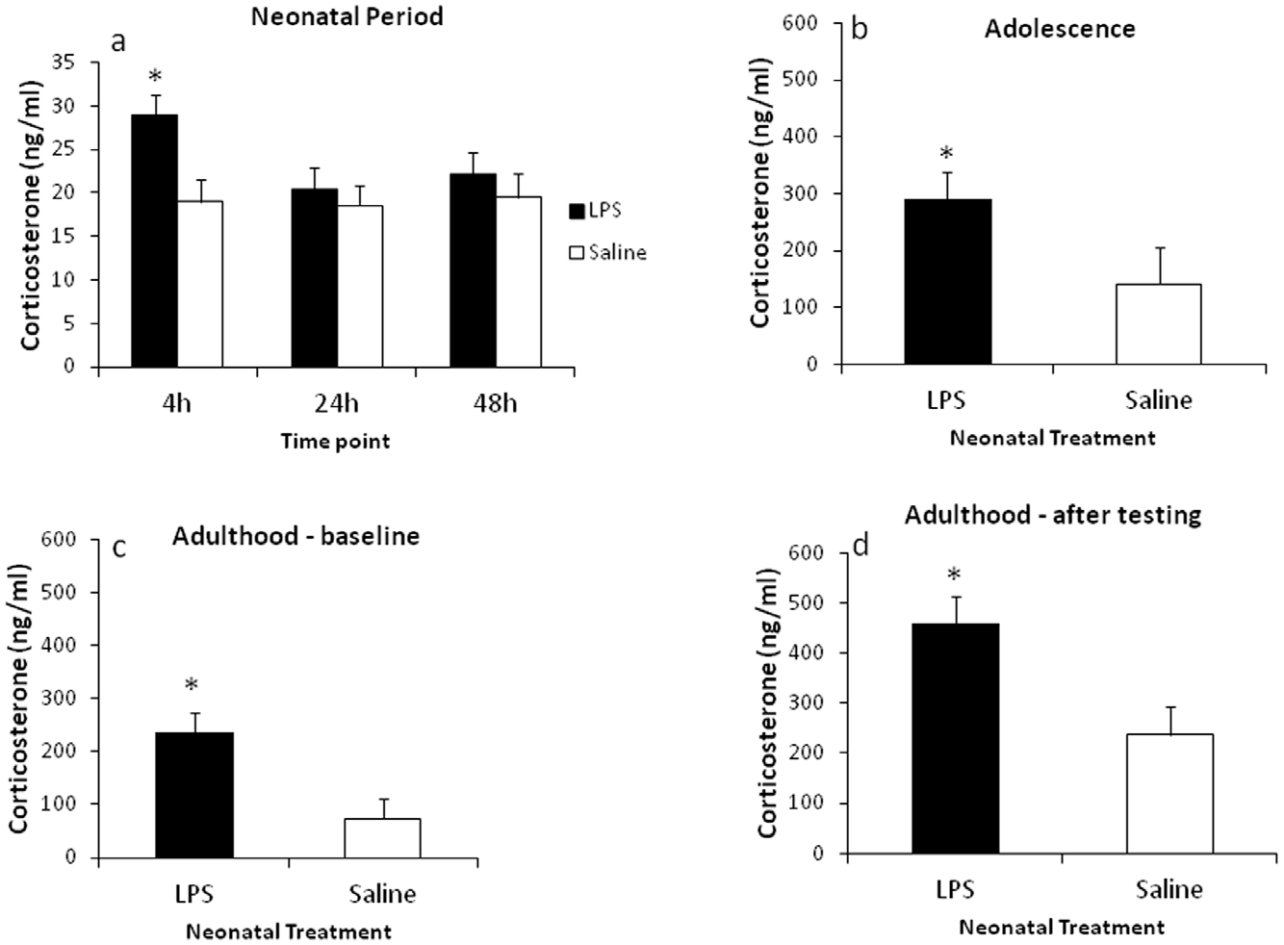
Effect of neonatal LPS exposure on plasma corticosterone levels. Corticosterone levels in neonatal period (PNDs 5-7) (A), in adolescence (PND 50) (B), and in adulthood (PND 85) (C). (D) Demonstrates changes in circulating corticosterone following the respiratory testing in adult rats (PND 85). Filled bars represent neonatally-treated LPS rats, hollow bars represent neonatally-treated saline controls. Values are mean±SEM. *p<.05.

Assessment of circulating corticosterone levels during the neonatal period revealed a significant effect of treatment (*F*_(8,26)_ = 4.09, *p*<.005), with LPS-treated males exhibiting significantly increased levels of circulating corticosterone (*M*  = 24.187, *SE*  = 1.436) than saline-treated controls (*M*  = 18.93, *SE*  = 1.463), and a significant interaction between the time point of blood collection and treatment (*F*_(10,26)_ = 3.88, *p*<.005). Planned comparisons revealed LPS-treated neonates had significantly increased corticosterone levels at 4 h following LPS challenge on PND 5 (*t*_(15)_  = 2.89, *p*<.05), with no differences at later time point, as shown in Fig. 2a.

LPS-treated males exhibited increased baseline corticosterone levels on PND 50 (*F*_(4,11)_ = 4.69, *p*<.05), and in adulthood, on PND 85 (*F*_(1,10)_ = 8.89, *p*<.05) as demonstrated in Fig. 2b and 2c. Significantly increased corticosterone levels were evident in LPS-treated males immediately following the respiratory testing (*F*_(1,13)_  = 6.12, *p* = .05), see Fig. 2d.

### Anxiety-like Behaviours

**Figure 3:**
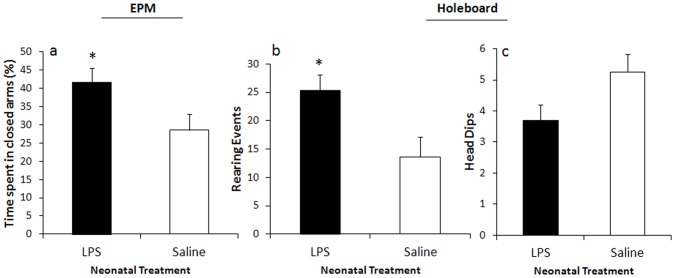
Effect of neonatal LPS exposure on anxiety-like behaviours in adulthood. Effect of neonatal LPS exposure on a percentage of time spent in the closed arms of the EPM (A), on a mean number of exploratory head dips (B) and risk assessment behaviours (C) in the holeboard apparatus. Filled bars represent neonatally-treated LPS rats, hollow bars represent neonatally-treated saline controls. Values are mean±SEM. *p<.05.

Neonatal LPS treatment resulted in significantly increased percentage of time spent in closed arms of the EPM, compared to saline-treated controls (*F*_(4,14)_  = 3.39, *p* = .05), Fig. 3a. Fig. 3b demonstrates that LPS-treated animals engaged in more risk assessment behaviour (rearing events) in the holeboard apparatus (*F*_(1,15)_ = 5.03, *p* = .05). Trends, which approached significance (*p* = .06), indicated that LPS-treated males exhibited fewer exploratory head dips in the holeboard apparatus than their saline-treated counterparts, Fig. 3c. No significant differences were observed in other measures.

### Gene Expression

**Figure 4:**
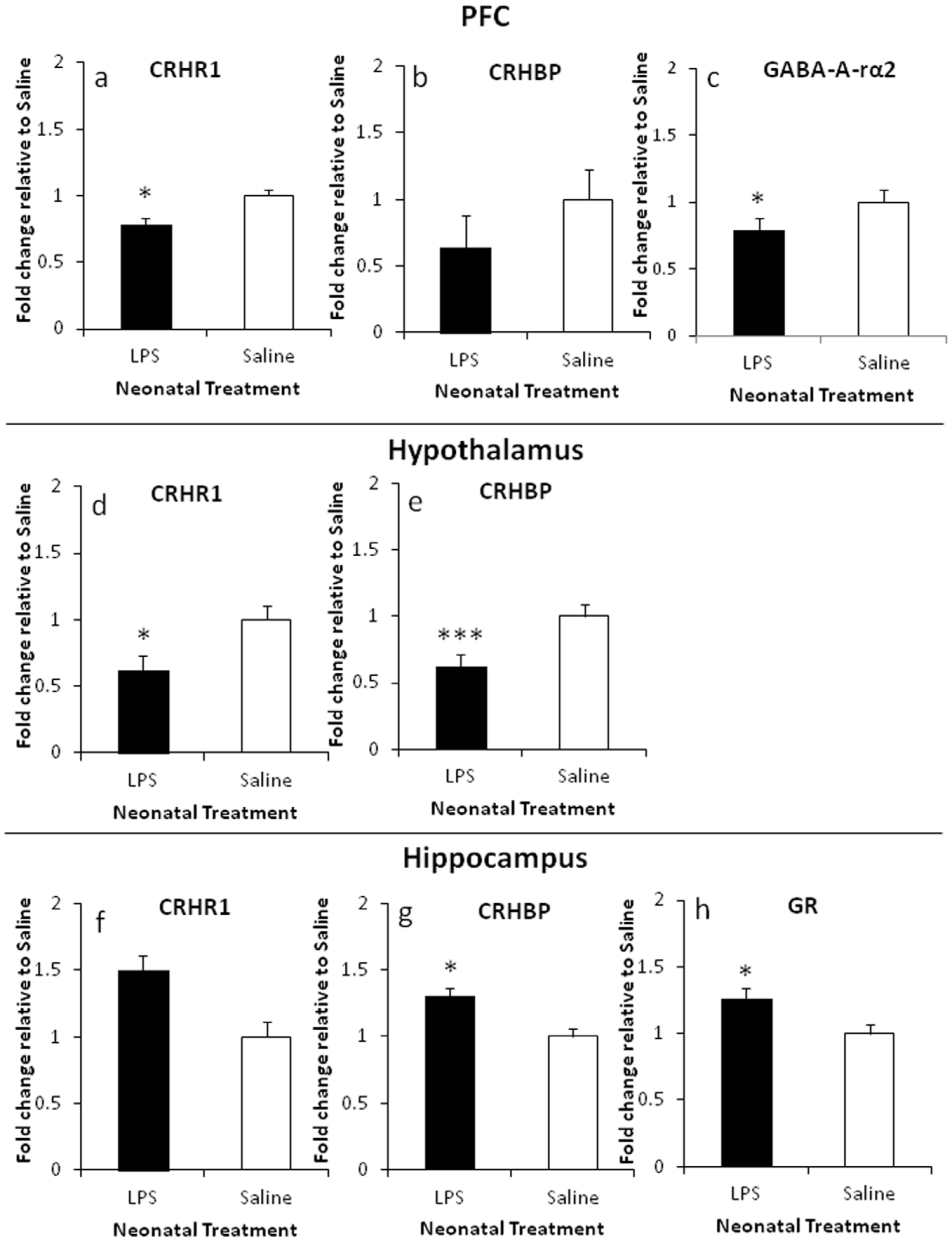
Gene expression in the PFC, the hypothalamus and the hippocampus, presented as a fold change relative to the saline control. (A–C) Represent changes in CRHR1, CRHBP and GABA-Arα2 mRNA levels in the PFC. (D–E) Represent changes in CRHR1 and CRHBP mRNA levels in the hypothalamus. (F–H) Represent changes in CRHR1, CRHBP and GR mRNA levels in the hippocampus. Values are mean±SEM. *p<.05, ***p<.001.

Significantly decreased CRHR1 mRNA levels were evident in LPS-treated males in the PFC and hypothalamus (*F*_(1,10)_ = 12.02, *p*<.05; *F*_(1,10)_ = 6.09, *p*<.05, Fig. 4a and 4d, respectively). However, in the hippocampus trends indicated that neonatal LPS exposure resulted in increased CRHR1 mRNA expression (*p* = .16), Fig. 4f. Similarly, CRHBP mRNA levels were decreased in the PFC of LPS-treated animals, approaching significance (*p* = .09), significantly decreased in the hypothalamus (*F*_(1,10)_ = 19.24, *p*<.001), but significantly increased in the hippocampal region (*F*_(1,10)_ = 6.04, *p*<.05), see Fig. 4b, 4e and 4g, respectively. Fig. 4c demonstrates that GABA-A-rα2 mRNA expression was significantly decreased in the PFC of LPS-treated males (*F*_(1,11)_ = 5.54, *p*<.05). Significantly increased GR mRNA levels were evident only in the hippocampus of LPS-treated animals (*F*_(1,11)_ = 6.25, *p*<.05), see Fig. 4h. No differences were observed in CRH and MR mRNA expression. Refer to Table 3 for the complete list of gene expression.

**Table 3:** Gene expression in the PFC, hypothalamus and hippocampus.

Target gene	PFC	Hypothalamus	Hippocampus
*CRH*	1.229 (±.166)	1.114 (+.214)	1.188 (+.119)
*CRHR1*	**−1.287 (**±**.049)** *	**−1.605 (+.108)** *	**1.496 (+.231)**
*CRHBP*	**−1.567 (**±**.144)**	**−1.602 (+.061)** ***	**1.304 (+.087)** *
*GABA-A-rα2*	**−1.269 (**±**.066)** *	**−**1.067 (+.137)	**−**1.025 (+.042)
*GR*	––	1.098 (+.064)	**1.262 (+.077)** *
*MR*	––	1.003 (+.115)	1.033 (+.078)

Data presented as a fold change relative to the saline control (mean±SEM).

* *p*<.05,

*** *p*<.001. See Fig. 4 for graphic representation of the highlighted values.

### TH Analysis

**Figure 5:**
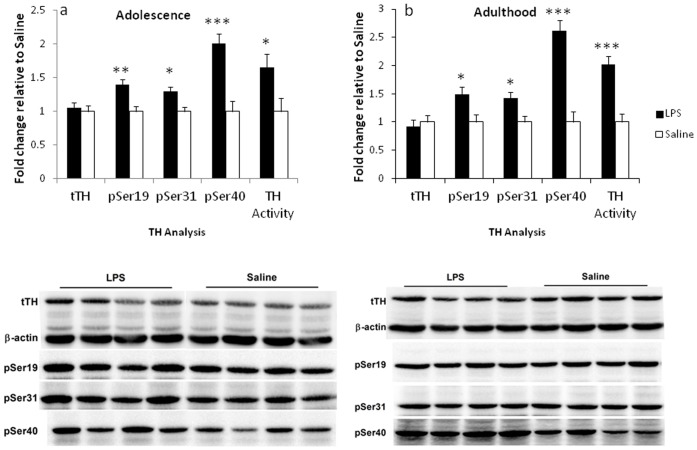
Effect of neonatal LPS exposure on TH protein, phosphorylation at Ser19, Ser31, Ser40 and TH activity levels. Changes in TH are expressed as a fold change relative to the saline control in the adrenal glands in adolescence (PND 50) (A) and adulthood (B). Representative immunoblots demonstrate the effect of LPS and saline treatments. Filled bars represent neonatally-treated LPS rats, hollow bars represent neonatally-treated saline controls. Values are mean±SEM. *p<.05, **p<.01, ***p<.001.

A significant effect of treatment was observed in regards to TH phosphorylation at all 3 serine residues (pSer19, pSer31 and pSer40) and TH activity in adolescent (*F*_(1,12)_ = 13.72, *p*<.005; *F*_(1,12)_ = 9.86, *p*<.05; *F*_(1,12)_ = 22.52, *p*<.0001; *F*_(1,12)_  = 5.36, *p*<.05, respectively, see Fig. 5a) and adult animals (*F*_(1,10)_ = 6.71, *p*<.05; *F*_(1,10)_ = 8.42, *p*<.05; *F*_(1,10)_ = 39.63, *p*<.001; *F*_(1,10)_  = 22.73, *p* = .001, respectively, see Fig. 5b). No significant difference in total TH protein levels was evident at any of the time points.

### Respiratory Testing

**Figure 6:**
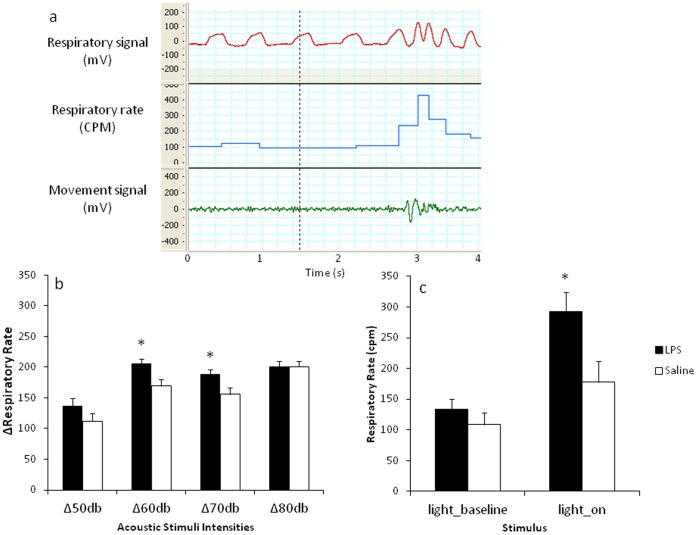
Effect of neonatal LPS exposure on respiratory rate in adulthood. (A) Representative raw data records of respiratory signal (mV), respiratory rate (cycles per minute (CPM)) and movement signal (mV) prior and following a sensory stimulus (broken vertical line). (B) Represents Δ changes from baseline (±SEM) in respiratory rate in response to acoustic stimuli as generated by the Change Detection model. (C) Represents changes (CPM±SEM) in respiratory rate prior and in response to light stimulus. Filled bars represent neonatally-treated LPS rats, hollow bars represent neonatally-treated saline controls. *p<.05.

Analysis of changes in respiration by using a Change Detection model revealed that LPS-treated males responded with a tendency to increase respiration when introduced to the stimulus of lowest intensity (50 dB). Despite that *t* test analysis revealed no significant difference in the magnitude of response between the LPS and saline-treated animals (*t_(12)_*  = 1.42, *p* = .18), Bayes factor (*B*_01_ = .97) constitutes an approximately equal likelihood for the preference for either null or the alternative hypothesis. In response to the 60 dB stimulus, LPS-treated animals responded with a significantly increased respiratory rate (*t_(15)_*  = 2.88, *p*<.05; *B*_01_ = .15). This difference from saline-treated controls remained significantly increased when presented with the 70 dB stimulus (*t_(15)_*  = 2.57, *p*<.05; *B*_01_ = .24). No differences were observed at the highest intensity (80 dB) between the neonatally treated groups as reflected in both statistical approaches (*t_(14)_*  = .005, *p = *.99; *B*_01_ = 2.18). See Fig. 6b.

As demonstrated in Fig. 6c, in response to light stimulus LPS-treated males exhibited an increased respiratory rate as compared to saline-treated controls (*F*_(4,15)_ = 3.61, *p*<.05), with no differences in baseline respiratory rate obtained prior to the presentation of the stimulus. See Fig. 6a for a representation of raw data records.

## Discussion

The primary aim of this study was to investigate the long term consequences of neonatal immune challenge on ANS functioning, and its association with behavioural and neuroendocrine indices in an anxiety-like phenotype. Here, we replicate and confirm this model by demonstrating increased anxiety-like behaviours and alterations to the HPA axis following neonatal LPS challenge. A significant outcome of this study is the demonstration that an exposure to LPS during the neonatal period is also associated with persistent alterations to ANS functioning. This is demonstrated using a behavioural index of ANS activity (via plethysmographic assessment of respiratory rate [29], [30]), and supported by biochemical analysis of adrenal TH phosphorylation, activity and protein levels. These findings suggest for the first time, to our knowledge, that neonatal LPS results in persistent alterations to autonomic activity - a crucial component in the biobehavioural response to stress.

LPS challenge in the neonate induces an acute increase in TH phosphorylation and activity in neonatal adrenals [13], [14], [25]. The current study importantly demonstrates that this increased activity and phosphorylation persists into adulthood. TH is the first rate-limiting enzyme in catecholamine biosynthesis [36]. Increased release of catecholamines is an essential aspect of the stress response, including inflammatory stress. The acute phase response to an inflammatory insult typically includes an immediate activation of the ANS both at the level of the parasympathetic and the sympathetic nervous systems [27]. Increased catecholamine release is then accompanied by a compensatory increase in catecholamine synthesis, in which TH plays an essential role, catalysing the hydroxylation of L-tyrosine to DOPA (reviewed in [36]). Under these conditions TH activity is normally inhibited. In response to stress, TH phosphorylation at serine residues disinhibits the catalytic domain and initiates enzymatic activity. While phosphorylation at three serine residues (Ser40, Ser 31 and Ser19) in the N-terminal domain of TH contributes to its activity, phosphorylation at Ser40 increases the enzymatic activity directly and to a greater extent than phosphorylation of the other sites [36], [37]. *In vitro* studies indicated that the acute phase response of TH activation occurs within minutes due to increased phosphorylation, and is then followed by a sustained phase response over several hours. During the chronic phase (over several days) this response is mediated by an increase in TH mRNA levels and a subsequent protein synthesis [37]. We confirmed this observation in *in vivo* studies through the demonstration that the sustained phase of TH activation in neonatal adrenal glands persists for up to 24 h after LPS administration [13], [25]. These changes were manifested by increased phosphorylation and activity of the enzyme, without alterations in protein levels. Increases in TH protein were evident only after 48 h, without a concomitant change in TH activity [25].

In the current study, increased phosphorylation at all three serine residues (Ser19, Ser31 and Ser40) and increased enzymatic activity were observed independent of changes in TH protein, in animals treated with LPS as neonates, at both the adolescent and adult time points. Previous *in vivo* studies have reported a disassociation between alterations in TH phosphorylation and activity, and TH protein levels in response to an acute [38] or chronic [39], [40] stressor, suggesting that sustained phosphorylation represents a distinct regulatory mechanism of catecholamine synthesis, independent of TH protein synthesis [37]. Therefore, our data suggests that neonatal exposure to LPS results in an initial and sustained phase of TH activation in the neonatal period, which is associated with a long lasting effect on TH activity, mediated by TH phosphorylation but not by protein synthesis.

Increased glucocorticoids, resulting from either exposure to stress or direct administration of synthetic glucocorticoids, increase TH activity in sites of catecholamine production, including the adrenal medulla [41]. A functional interdependence between the adrenal cortical and medullary systems [42] suggests that the increased TH activity evident in the current study could potentially be triggered by increased and prolonged exposure to glucocorticoids. However, since our previous data [13], [25] indicate that in neonates TH activity is more extended than the corticosterone response to LPS treatment, it appears more likely that an immune challenge during the neonatal period independently activates the HPA axis and the ANS, resulting in developmental programming of both systems.

Given that the TH analysis has indicated long term alterations in autonomic activity, we aimed to validate this observation using a behavioural paradigm that specifically targets autonomic function. Using a recently established model of respiratory testing, which assesses respiratory responses to mild sensory stimuli, the current study assessed respiration in rats as a measure of arousal. Previous studies have demonstrated that this behavioural measure is a valid, sensitive and reliable index of rapid changes in autonomic activity [29], [30]. A recent study using a similar respiratory testing procedure indicated that systemic administration of an anxiolytic drug substantially reduces respiratory responses to acoustic stimuli and restraint stress [43], suggesting that assessment of respiratory rate in rodents represents a distinct measure of anxiety states. Moreover, due to the rapidity of change, respiratory responses are argued to be more sensitive than changes in cardiovascular parameters, providing an efficient temporal resolution, similar to EEG desynchronization, for measuring ANS activity [30]. As such, a simultaneous assessment of heart rate and respiratory parameters in response to short-lasting acoustic stimuli (60 dB to 90 dB) revealed that while respiratory responses typically show intensity-dependent increases, heart rate responses to the same intensities of the stimuli are less consistent and demonstrate no obvious trend [29], [30]. The lack of a significant increase in heart rate responses in these previous studies was attributed to the relatively low intensity of the stimuli, suggesting an insensitivity of heart rate to subtle changes in arousal [29]. In the current study LPS-treated rats, when tested in adulthood, exhibited significantly increased respiratory rates at acoustic intensities that were several fold lower than those generating the same magnitude of response in saline-treated controls. This finding was replicated in response to bright light, and as such indicated that LPS-treated animals, when tested in adulthood, responded more intensely in terms of respiratory rate when exposed to visual and auditory stimuli.

In the current study, alterations in respiratory responses exhibited by the LPS-treated animals were associated with increases in anxiety-like behaviours and HPA axis activity. This is consistent with previous reports from this laboratory [4], [6], [13], [15]. Therefore to extend these findings we investigated the expression of genes implicated in the aetiology of anxiety in the limbic regions, involved in the regulation of endocrine and autonomic stress-related responses, and responsible for the anxiety-related behavioural changes [22]. A significant reduction in GABA-Arα2 mRNA levels was evident in the PFC of LPS-treated animals. Given the major role of the α2 GABA-A receptor subtype in mediating anxiolytic effects of benzodiazepines, this is consistent with the reported anxiety-like phenotype in the current and other studies [4], [6], [13], [15]. Moreover, GABAergic innervation of the PVN provides an inhibitory tone, modulating the HPA axis and autonomic activity. Within the PFC, GABAergic interneurons regulate the activity of glutamatergic neurons [44]. Reduced GABA-mediated inhibition, achieved by blockade of cortical GABA-A receptors, has been previously reported to result in cognitive deficits in schizophrenia models [45], [46]. Although forebrain limbic structures do not directly innervate the PVN, inhibitory input from these limbic areas is received via intermediate synapses, inhibiting the HPA axis and autonomic responses to stress (reviewed in [22]). Whilst only one subunit of the GABA-A receptor system was examined in this study, this finding suggests a possible reduction in GABAergic content in the PFC of LPS-treated males, which may subsequently result in reduced GABA-mediated inhibition of cortical glutamatergic pyramidal neurons and lead to increased HPA axis activity and increased sympathetic response. In addition, further distinct regional assessment of GABA functioning is required. Nevertheless, this data is of a particular importance, given that neonatal LPS stimulus coincided with a critical period of functional maturation of the GABAergic synapses. Specifically, during the first week of life GABAergic plasticity in the neonatal rat hippocampus can be induced by postsynaptic activity of pyramidal cells [47]. Whether the peripheral immune challenge results in synaptic activity, which may induce long-term plasticity at inhibitory synapses, is yet to be elucidated.

Examination of CRHR1 and CRHBP mRNA levels revealed substantial downregulation of gene expression in the PFC and the hypothalamus, but upregulation in the hippocampus of LPS-treated animals. The CRH system has a regulatory role in HPA and ANS functioning [22]. While CRH exerts its actions via two receptors subtypes (CRHR1 and CRHR2), it binds with higher affinity to CRHR1 [48], and CRHBP regulates availability of CRH to its receptors [49]. Regional-specific expression and signalling capacity of CRH and its receptors has implications for anxiety and depression disorders [49]. While CRH deficiency has been reported to result in impaired HPA axis activity with no behavioural effects [50], CRHR1 depletion in mice has been associated with a decrease in anxiety-like behaviours [51], suggesting a differential importance of the receptor and its ligand in the regulation of stress-induced activity. A selective model of postnatal deletion of CRHR1 in the forebrain, introduced by Müller and colleagues, revealed a distinct role of CRH/CRHR1 neuronal pathways involved in behavioural regulation from those regulating HPA axis activity [52], with deletion of CRHR1 in limbic structures leading to aberration of anxiety-like behaviours independent of the HPA axis functioning. It implies, therefore, that CRHR1 is essential in mediating the behavioural responses to stress.

CRHBP, together with CRH receptors, mediates the activity of CRH in the brain and the pituitary, presumably acting as a negative regulator of CRH. CRHBP expression can be modulated by glucocorticoids and is cell-dependent, with increased pituitary levels in response to stress, suggesting its role in a negative feedback on the HPA axis [53]. Models of CRHBP overexpression and deficiency demonstrated an intricacy of homeostatic mechanisms regulating the HPA axis activity, with no changes in basal and adrenocorticotropic hormone (ACTH) levels in these models, but with a blunted ACTH response to the LPS stimulus in mice overexpressing CRHBP centrally and peripherally, suggesting a compensatory increase or decrease of CRH may occur in response to alterations in CRHBP levels (reviewed in [53]).

A model of HPA axis dysfunction in depression and anxiety, proposed by Reul and Holsboer (2002) [54], suggests that the HPA hyperactivity may derive from the decreased negative inhibitory feedback, normally exerted by the hippocampal structures on the hypothalamus, but an increased effect of the central nucleus of amygdala, stimulating further activation of the HPA axis. Therefore the regional variability in the expression of both CRHR1 and CRHBP in the current study may be a result of prolonged exposure to circulating glucocorticoids, leading to diminished ability of the hippocampus to exert an inhibitory tone on the hypothalamic parvocellular neurons and potentially a compensatory alteration of CRHR1 and CRHBP mRNA levels in the PFC and the hypothalamus. Neonatal LPS exposure was previously found to result in increased mRNA expression of CRH in the PVN as well as increased median eminence levels of CRH and arginine vasopressin in adult animals neonatally treated with LPS [11]. In addition, increase in CRH mRNA content in the PVN was reported in 1-month-old mice, neonatally treated with LPS [55]. While this effect was not evident in the current study, we have assessed the mRNA content of the whole hypothalamic and hippocampal extracts. Therefore, examination of subregions may provide an insight on the regional-specific gene expression. Nevertheless, here we demonstrate the programming effect of neonatal LPS challenge on other components of the CRH system, which play an unequivocal role in regulation of the ANS, HPA axis activity and anxiety-like behaviours.

Increased expression of GR mRNA content was found in the hippocampus of LPS-treated animals. Previously, neonatal LPS treatment has been reported to decrease GR binding and density in the hippocampus, hypothalamus and frontal cortex of adult animals, adrenalectomized prior to brain collection [11]. The discrepancy between our findings and the latter may reflect differences in the functional aspects of GRs measured, since the current study assessed mRNA levels in animals with intact adrenals. In support of the current trend, increased hippocampal expression of GR mRNA, and no changes in MR mRNA levels, have been previously demonstrated in a similar model of neonatal LPS exposure in mice, when assessed during the second week of life [55]. In addition, contradictory findings have been reported, whereby increased glucocorticoid levels have been associated with either increased [56], [57], decreased [58], [59], or unchanged [60] GR binding or mRNA levels in the hippocampus. It is also plausible that elevated levels of circulating corticosterone, in conjunction with increased expression of GR in the hippocampus may constitute a state of glucocorticoid resistance, commonly present in inflammatory and neuropsychiatric disorders [61], accompanied by an altered balance of GR and MR expression, contributing to the increased emotional arousal [62].

### Conclusions

Our findings suggest that exposure to an immune challenge in the early postnatal period of life drives long term alterations in the neuroendocrine and autonomic systems, and is associated with alterations in behavioural functioning, overall indicative of an anxiety phenotype. While programming of the HPA axis activity and anxiety-like behaviours by neonatal LPS exposure has been previously demonstrated, this study provides novel insights into the effects of an early life immune challenge on ANS functioning. Since prolonged exposure to increased endocrine and autonomic activity is a known epidemiological risk factor for development of metabolic disorders, such as visceral obesity and insulin resistance, as well as cardiovascular diseases [63], our study further points to the significance of alterations in the neonatal microbial environment on long term physiological outcomes. Whether the functional changes in the ANS are induced directly by the neonatal LPS exposure or mediated via the hyperactive HPA axis is yet to be determined, however, alterations in specific gene expression in the brain in mutual control regions suggests concurrent programming of physiological systems may exist.
